# The impact of Ty3-*gypsy *group LTR retrotransposons *Fatima *on B-genome specificity of polyploid wheats

**DOI:** 10.1186/1471-2229-11-99

**Published:** 2011-06-03

**Authors:** Elena A Salina, Ekaterina M Sergeeva, Irina G Adonina, Andrey B Shcherban, Harry Belcram, Cecile Huneau, Boulos Chalhoub

**Affiliations:** 1 Institute of Cytology and Genetics, Siberian Branch of the Russian Academy of Science, Lavrentieva ave. 10, Novosibirsk, 630090, Russia; 2 UMR INRA 1165 - CNRS 8114 UEVE - Unite de Recherche en Genomique Vegetale (URGV), 2, rue Gaston Cremieux, CP5708, 91057 Evry cedex, France

## Abstract

**Background:**

Transposable elements (TEs) are a rapidly evolving fraction of the eukaryotic genomes and the main contributors to genome plasticity and divergence. Recently, occupation of the A- and D-genomes of allopolyploid wheat by specific TE families was demonstrated. Here, we investigated the impact of the well-represented family of *gypsy *LTR-retrotransposons, *Fatima*, on B-genome divergence of allopolyploid wheat using the fluorescent *in situ *hybridisation (FISH) method and phylogenetic analysis.

**Results:**

FISH analysis of a BAC clone (BAC_2383A24) initially screened with Spelt1 repeats demonstrated its predominant localisation to chromosomes of the B-genome and its putative diploid progenitor *Aegilops speltoides *in hexaploid (genomic formula, BBAADD) and tetraploid (genomic formula, BBAA) wheats as well as their diploid progenitors. Analysis of the complete BAC_2383A24 nucleotide sequence (113 605 bp) demonstrated that it contains 55.6% TEs, 0.9% subtelomeric tandem repeats (Spelt1), and five genes. LTR retrotransposons are predominant, representing 50.7% of the total nucleotide sequence. Three elements of the *gypsy *LTR retrotransposon family *Fatima *make up 47.2% of all the LTR retrotransposons in this BAC. *In situ *hybridisation of the *Fatima*_2383A24-3 subclone suggests that individual representatives of the *Fatima *family contribute to the majority of the B-genome specific FISH pattern for BAC_2383A24. Phylogenetic analysis of various *Fatima *elements available from databases in combination with the data on their insertion dates demonstrated that the *Fatima *elements fall into several groups. One of these groups, containing *Fatima*_2383A24-3, is more specific to the B-genome and proliferated around 0.5-2.5 MYA, prior to allopolyploid wheat formation.

**Conclusion:**

The B-genome specificity of the *gypsy*-like *Fatima*, as determined by FISH, is explained to a great degree by the appearance of a genome-specific element within this family for *Ae. speltoides*. Moreover, its proliferation mainly occurred in this diploid species before it entered into allopolyploidy.

Most likely, this scenario of emergence and proliferation of the genome-specific variants of retroelements, mainly in the diploid species, is characteristic of the evolution of all three genomes of hexaploid wheat.

## Background

Transposable elements (TEs) of various degrees of reiteration and conservation constitute a considerable part of wheat genomes (80%). TEs are a rapidly evolving fraction of eukaryotic genomes and the main contributors to genome plasticity and divergence [1,2]. Class I TEs (retrotransposons) are the most abundant among the plant mobile elements, constituting 19% of the rice genome and at least 60% of the genome in plants with a larger genome size, such as wheat and maize [3-6]. In wheat, the majority of class I TEs are LTR (long terminal direct repeats) retrotransposons [7,8]. The internal region of LTR retrotransposons contains *gag *gene, encoding a structural protein, and polyprotein (*pol*) gene, encoding aspartic proteinase (AP), reverse transcriptase (RT), RNase H (RH), and integrase (INT), which are essential to the retrotransposon life cycle [9,10]. Because of their copy-and-paste transposition mechanism, retrotransposons can significantly contribute to an increase in genome size and, along with polyploidy, are considered major players in genome size variation observed in flowering plants [11-13].

Genomic *in situ *hybridisation (GISH) provides evidence for TEs involvement in the divergence between genomes. GISH, a method utilising the entire genomic DNA as a probe, makes it possible to distinguish an individual chromosome from a whole constituent subgenome in a hybrid or an allopolyploid genome. Numerous examples of successful GISH applications in the analysis of hybrid genomes have been published, including in allopolyploids, lines with foreign substituted chromosomes, and translocation lines [14-17]. It is evident that the TEs distinctively proliferating in the genomes of closely related species are the main contributors to the observed differences detectable by GISH.

GISH identification of chromosomes in an allopolyploid genome depends on the features specific during the evolution of diploid progenitor genomes to the formation of allopolyploid genomes and further within the allopolyploid genomes. Three events can be considered in the evolutionary history of hexaploid wheats. The first event led to the divergence of the diploid progenitors of the A, B and D genomes from their common ancestors more than 2.5 million years ago (MYA). The next event was the formation of the allotetraploid wheat (2*n *= 4*x *= 28, BBAA) less than 0.5-0.6 MYA. Hexaploid wheat (*2n *= 6*x *= 42, BBAADD) formed 7,000 to 12,000 years ago [18-21]. It is considered that *Triticum urartu *was the donor of the A genome; *Aegilops tauschii *was donor of the D genome; and the closest known relative to the donor of the B genome is *Aegilops speltoides*.

GISH using total *Ae. tauschii *DNA as a probe has demonstrated that the chromosomes of the D genome, which was the last one to join the allopolyploid genome, are easily identifiable, and the hybridisation signal uniformly covers the entire set of D-genome chromosomes [22]. Hybridisation of total *T. urartu *DNA to *Triticum dicoccoides *(genomic formula, BBAA) metaphase chromosomes distinctly identifies all A-genome chromosomes [23]. All these facts suggest the presence of A- and D-genome specific retroelements. Construction of BAC libraries for the diploid species with AA (*Triticum monococcum*) and DD (*Ae. tauschii*) genomes allowed these elements to be identified. Fluorescent *in situ *hybridisation (FISH) of BAC clones made it possible to select the clones giving the strongest hybridisation signal that was uniformly distributed over all chromosomes of the A or D genomes of hexaploid wheat [24]. Subcloning and hybridisation have demonstrated that the TEs present in these BAC clones may determine the observed specific patterns. It has been also shown that A-genome-specific sequences have high homology to the LTRs of the *gypsy*-like retrotransposons *Sukkula *and *Erika *from *T. monococcum*. The D-genome-specific sequence displays a high homology to the LTR of the *gypsy*-like retrotransposon *Romani *[24].

The GISH pattern of the B-genome chromosomes is considerably more intricate. The total *Ae. speltoides *DNA used as a probe allowed the B-genome chromosomes to be identified in the tetraploid wheat *T. dicoccoides; *however, the observed hybridisation signal was discrete, i.e., it did not uniformly cover all of the chromosomes but rather was concentrated in individual regions [22,23]. Such a discrete hybridisation signal suggests the presence of genome-specific tandem repeated DNA sequences. It has been shown that a characteristic of the B genome is the presence of GAA satellites [25] and several other tandem repeats [26], which are either absent or present in a considerably smaller amount in the A- and D-genomes. A more intensive hybridisation to individual regions of B-genome chromosomes as compared with the A genome was also demonstrated for the probe for Ty1-*copia *retroelements [27]. The existence of B-genome specific retrotransposons analogous in their chromosomal localisation to those detected for the A and D genomes can be only hypothesised.

Another intriguing issue is the time period when TEs most actively proliferated in the wheat genomes. An increase in the number of determined DNA sequences from the wheat A and B genomes gave the possibility to date the insertion of TEs in these two genomes. The majority of TEs differential proliferation in the wheat A and B genomes (83 and 87%, respectively) took place before the allopolyploidisation event that brought them together in *T. turgidum *and *T. aestivum*. Allopolyploidisation is likely to have neither positive nor negative effects on the proliferation of retrotransposons [6].

The data on TEs insertions in orthologous genomic regions are not contradictory to the above results on TEs proliferation in diploid progenitors that occurred before allopolyploidisation. A comparison of orthologous genomic regions demonstrates the absence of conserved TEs insertions in *T. urartu*, *Ae. speltoides*, and *Ae. tauschii*, which are putative diploid donors to hexaploid wheat [21,28-31]. On the contrary, a comparison of orthologous regions in the diploid genomes and the corresponding subgenomes of polyploid wheat species suggests the presence of conserved TEs insertions [29,30,32]. However, note that the intergenic space, composed mainly of TEs, may be subject to an extremely high rate of TEs turnover [33]. In particular, analysis of the intergenic space in the orthologous *VRN2 *loci of *T. monococcum *and the A genome of tetraploid wheat has demonstrated that 69% of this space has been replaced over the last 1.1 million years [34]. All this suggests intensive processes of TEs proliferation and turnover in the diploid progenitors of allopolyploid wheat.

Thus, it is reasonable to expect that the B genome contains specified retrotransposons dispersed over all constituent chromosomes that proliferated as early as in the diploid progenitor of this genome.

We have previously analysed nine BAC clones of *T. aestivum *(genomic formula, BBAADD) cv. Renan and identified BAC clone 2383A24 as hybridising to a number of chromosomes [35] in a dispersed manner. In this work, we have shown a predominant localisation of BAC_2383A24 to the B-genome chromosomes of common wheat and comprehensively analysed its sequence, which gives the background for clarifying the reasons underlying its B-genome specificity. The contribution of the LTR retrotransposon *Fatima*, the most abundant element in this clone, to the B-genome specificity of polyploid wheat and the divergence of common wheat diploid progenitors were studied.

## Results

### BAC-FISH with the chromosomes of *Triticum *allopolyploids and their diploid relatives

**Figure 1:**
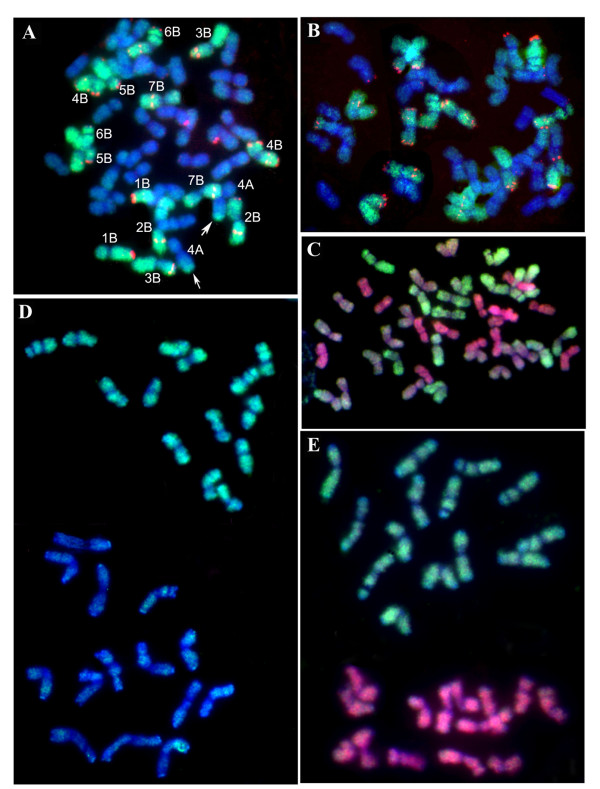
**FISH of mitotic metaphase chromosomes of *Triticum *and *Aegilops *species**. The species analysed are (a-c) *T. aestivum *cv. Chinese Spring; (d) *Ae. speltoides *and *T. urartu*; (e) *Ae. speltoides *and *Ae. tauschii*. The probe combinations are: (A, C-E) BAC clone 2383A24 (green); (B) 2383A24/15 (green); (A and B) pSc119.2 (red); (C and E) *Ae. tauschii *DNA (red). Arrows point to the translocation of 7BS to 4AL.

BAC-FISH was performed with the allopolyploid wheats *T. durum *(genomic formula, BBAA) and *T. aestivum *(genomic formula, BBAADD) as well as their diploid progenitors, including the donor of the A-genome, *T. urartu*, donor of the D-genome, *Ae. tauschii*, and the putative donor of the B-genome, *Ae. speltoides*. The chromosomal localisation of BAC_2383A24 in the allopolyploid species was determined by simultaneous *in situ *hybridisation using the probe combinations pSc119.2 + BAC and pAs1 + BAC. The pSc119.2 and pAs1 are tandem repeats that are used as probes for wheat chromosome identification [36]. Figure 1A shows the hybridisation pattern for *T. aestivum *(cv. Chinese Spring) with the probes pSc119.2 and BAC_2383A24. The strongest hybridisation signals for BAC_2383A24 were on the 14 chromosomes of the *T. aestivum *B-genome. Analogous results were obtained for the remaining two analysed common wheat cultivars, Renan and Saratovskaya 29 (data not shown). In addition, using BAC_2383A24 as a probe, we succeeded in visualising the translocation of the 7B short-arm to the long-arm of the 4A chromosome (Figure 1A), which took place during the evolution of Emmer allopolyploid wheat [37,38]. The BAC-FISH experiments showed preferential BAC_2383A24 hybridisation to the B-genome chromosomes in the tetraploid species *T. durum *(genomic formula, BBAA, data not shown). Thus, the BAC_2383A24 probe can efficiently identify chromosomes from the B-genome of tetraploid and hexaploid wheat.

We also showed that the three genomes of common wheat (*T. aestivum*) can be identified using simultaneous *in situ *hybridisation with BAC_2383A24 and labelled genomic DNA of *Ae. tauschii*. In these experiments, the B-genome intensively hybridised with BAC_2383A24 (green color), the D-genome intensively hybridised with *Ae. tauschii *DNA (red color), and the A-genome displayed weak or no hybridisation with both probes (Figure 1C). The genome of *Ae. speltoides *is easily distinguishable by *in situ *hybridisation with BAC_2383A24 in the slide containing the metaphase chromosomes of both *Ae. speltoides *and *T. urartu *(Figure 1D). More contrasting distinctions are observed when BAC_2383A24 and *Ae. tauschii *DNA are simultaneously hybridised to the slides containing mixtures of the genomes of *Ae. speltoides *and *Ae. tauschii *(Figure 1E).

### Analysis of the nucleotide sequence of the B-genome-specific BAC clone 2383A24

To precisely determine the range of sequences that could possibly contribute to the B-genome specificity of the BAC_2383A24 FISH pattern, this BAC clone was sequenced and annotated (the corresponding data were deposited in GenBank under the accession number [GenBank: GU817319]).

**Figure 2:**
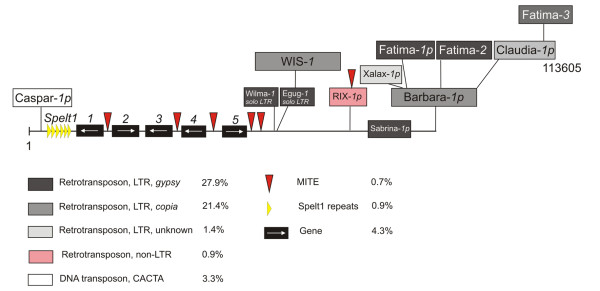
**Structural organisation of 113 605-bp *T. aestivum *genomic region marked by Spelt1 subtelomeric repeats**. The genomic region contains B-genome specific *Fatima *sequences (*p *at the ends of the names of transposable elements indicates that the corresponding elements are truncated).

Transposable elements constitute 55.6% of BAC_2383A24 (Table 1), and retrotransposons (class I) are the most abundant, constituting 51.6% of BAC_2383A24. LTR retrotransposons were also nest-inserted in each other (Figure 2). The most abundant family in the LTR retrotransposons for this BAC clone contains the *gypsy*-like *Fatima *elements (Table 1). BAC_2383A24 contains three copies, namely, *Fatima*_2383A24-1p (p indicates the elements with truncated ends), *Fatima*_2383A24-2, and *Fatima*_2383A24-3, which account for 47.2% of all LTR retrotransposons in this clone.

**Table 1:** The elements identified in the *T. aestivum *BAC clone 2383A24 (length, 113 605 bp).

Class, order, superfamily, family	Copy number	Sequence length, bp	Fraction in complete BAC_2383A24 sequence, %
**Class I elements (Retrotransposons)**	**11**	**58 604**	**51.6**

**LTR retrotransposons**	**10**	**57 590**	**50.7**

***gypsy***	**6**	**31708**	**27.9**

RLG_*Egug*_2383A24_solo_LTR	1	1503	

RLG_*Wilma*_2383A24_solo_LTR	1	1490	

RLG_*Sabrina*_2383A24-1p	1	1505	

RLG_*Fatima*_2383A24-1p, -2, and -3	3	27 210	

***copia***	**3**	**24 316**	**21.4**

RLC_*WIS*_2383A24-1	1	8353	

RLC_*Barbara*_2383A24-1p	1	6384	

RLC_*Claudia*_2383A24-1p	1	9579	

**Unknown LTR retrotransposons **RLX_*Xalax*_2383A24-1p	**1**	**1566**	**1.4**

**Non-LTR retrotransposons **LINE, RIX_2383A24-1p	**1**	**1014**	**0.9**

**Class II elements (DNA transposons)**CACTA, DTC_*Caspar*_2383A24-1p	**1**	**3693**	**3.3**

**MITE**	**6**	**747**	**0.7**

**Other known repeats**Spelt1 tandem repeats	**6**	**1010**	**0.9**

**Genes**	**5**	**4913**	**4.3**

**Unassigned sequences**			**39.2**

The copy number, total sequence length, and its percent content in the complete BAC_2383A24 sequence are shown for genes, the Spelt1 tandem repeat, and each TE class, order and superfamily.

The class II DNA transposons are represented by a single copy of the *Caspar*_2383A24-1p element, constituting only 3.3% of BAC-2383A24. Note that *Caspar*_2383A24-1p has a 95% identity over the entire sequence length to the *Caspar*_2050O8-1 element, which, according to our data, is characteristic of wheat subtelomeric regions [35,39]. *Caspar*_2383A24-1p is truncated at the 3'-end and contains the sequence that codes for transposase. The five hypothetical genes identified in BAC_2383A24 account for 4.3% of the entire BAC sequence (Table 2, Figure 2). Two hypothetical genes (2383A24.1 and 2383A24.3) contain transferase domains (Pfam PF02458), and their hypothetical protein products display an 88% identity to each other. Gene 2383A24.2 is located between the two transferase-coding genes and is very similar (80% identity) to the *Hordeum vulgare *tryptophan decarboxylase gene [GenBank: BAD11769.1]. The functions of the remaining two hypothetical genes, 2383A24.4 and 2383A24.5, have not yet been identified. However, they display significant similarity (>57% identity over >83% of their lengths) to hypothetical rice protein and display high similarity to one another (over 80% identity) in both nucleotide and amino acid sequences (Table 2). Thus, the five genes form a gene island of 23,670 bp located 9,737 bp from the 5'- end (Figure 2). The intergenic regions contain four MITE insertions and a 3 kb region similar to *T. aestivum *chloroplast DNA. Note that the 5'-end of this gene island contains a direct duplication of genes 2383A24.1 and 2383A24.3, which are similar to gene Os04g0194400, located on rice chromosome 4. However, the 3'-end carries an inverted duplication of genes 2383A24.4 and 2383A24.5, which are similar to gene Os01g0121600, localised to a distal region (1.22 Mb from the end) on the short arm of rice chromosome 1.

**Table 2:** The genes identified in non-TE and nonrepeated sequences of BAC_2383A24.

Identified genes	Hypothetical function	Positions in 2383A24	Protein length, residues	Support level
2383A24.1	Conserved hypothetical, transferase domain containing	9737 to 11 026	429	Similar to rice Os04g0194400 (58% identity, 100% coverage) Os04g0175500 (58% identity, 99% coverage EST support: +

2383A24.2	Putative decarboxylase protein	14 749 to 16 257	502	Similar to rice Os08g0140300 (79% identity, 100% coverage), to barley BAD11769.1 tryptophan decarboxylase (80% identity, 100% coverage) EST support: +

2383A24.3	Conserved hypothetical, transferase domain containing	19 745 to 21 019	424	Similar to rice Os04g0194400 (59% identity, 100% coverage) Os04g0175500 (59% identity, 99% coverage) EST support: +

2383A24.4	Unknown	26 839 to 27 333	164	Similar to rice Os01g0121600 (57% identity, 83% coverage) EST support: +

2383A24.5	Unknown	33 063 to 33 407	114	Similar to rice Os01g0121600 (73% identity, 88% coverage) EST support: +

BLAST alignments of the BAC_2383A24 sequence and the contigs containing mapped wheat ESTs (expressed sequence tags) from GrainGenes database [http://wheat.pw.usda.gov/GG2/blast.shtml] none identified any homology to BAC_2383A24 sequence.

BAC_2383A24 contains an array of six tandem subtelomeric Spelt1 repeats (five copies are each 177 bp long, and one copy is truncated to 125 bp). They constitute 0.9% of the clone length (Table 1, Figure 2). The presence of the Spelt1 tandem repeat and a *Caspar *element homologous to *Caspar*_2050O8-1 suggests the BAC_2383A24 clone likely originated from a subtelomeric chromosomal region [35].

We used Insertion Site-Based Polymorphism (ISBP) for developing a BAC_2383A24 specific TE-based molecular marker [40]. ISBP exploits knowledge of the sequence flanking a TE to PCR amplify a fragment spanning the junction between the TE and the flanking sequence. We selected one primer pair for the junction between the elements *Barbara*_2383A24-1p and *Fatima*_2383A24-2 (BarbL and BarbR). The primers BarbL/BarbR were used for localising BAC_2383A24 to the chromosomes of *T. aestivum *cv. Chinese Spring. PCR analysis using nullitetrasomic lines has demonstrated that the BarbL/BarbR fragment with a length of 1008 bp corresponding to BAC_2383A24 is characteristic of the 3B chromosome (see Additional File 1). The data on the homology between the DNA and amino acid sequences of 2383A24.4 and 2383A24.5 to the distal region of the rice 1S chromosome, which is syntenic to the short arm of wheat homoeologous group 3 chromosomes [41], also confirm this localisation (Table 2).

Note that characteristic of BAC_2383A24 is a higher gene density (one gene per 23 kb) relative to an average level of one gene per 100 kb, typical of wheat genome, and a lower TE content (55.6%) as compared with the mean TE level (about 80%) [6-8]. Analysis of the contigs along the 3B chromosome has demonstrated an increase in the gene density towards the distal chromosomal regions as well as a decrease in the TE content in these regions [8]. The contig ctg0011 on the distal region of the 3B short arm [8], whereto according to our data BAC_2383A24 is localized, displayed the most pronounced contrast with the average gene density values and TE contents of the wheat genome.

### The *gypsy*-like *Fatima *retrotransposon sequences are responsible for specific hybridisation to the B-genome

**Figure 3:**
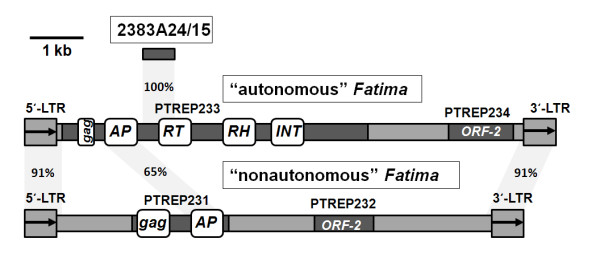
**The comparison of "autonomous" and "nonautonomous" variants of *Fatima***. The "autonomous" variant TREP3189 presented by the consensus nucleotide sequence, with the two open reading frames corresponding to hypothetical proteins PTREP233 (polyprotein) and PTREP234. The "nonautonomous" variant TREP3198 presented by the consensus nucleotide sequence, with the open reading frames corresponding to hypothetical proteins PTREP231 (polyprotein) and PTREP232. The conservative domains are indicated as follows: AP - aspartic proteinase, RT - reverse transcriptase, RH - RNAse H, INT - integrase, and gag - structural core protein. The conserved regions between "autonomous" and "nonautonomous" variants are indicated with light grey shading and the percent of homology is defined. Likewise the relative position of probe BAC2383A24/15 in reference to "autonomous" *Fatima *variant is marked with light grey; in "nonautonomous" variant, the sequence corresponding to BAC2383A24/15 is absent.

To detect the specific sequences that account for the major contribution to B-genome specific hybridisation, we subcloned BAC_2383A24. We subsequently screened subclones that gave a strong hybridisation signal with *Ae. speltoides *genomic DNA and selected several for further characterisation. Using the 435-bp subclone (referred to as 2383A24/15) as a probe for *in situ *hybridisation (Figure 3), we obtained B-genome specific signal distributions on the *T. aestivum *chromosomes similar to the initial BAC_2383A24 clone (Figure 1B). Sequence analysis of subclone 2383A24/15 shows that it corresponds to a region of the *Fatima*_2383A24-3 coding sequence and displays 85% sequence identity to the *Fatima*_2383A24-2 element; it has no matches with the *Fatima*_2383A24-1p element.

We failed to obtain B-genome specific hybridisation with different subclones corresponding to either other TEs or sequences in BAC_2383A24. Overall, our analysis suggests that the *gypsy*-like LTR retrotransposon *Fatima_*2383A24-3 is responsible for the B-genome specificity of BAC_2383A24 FISH.

### Phylogenetic analysis of the gypsy-like LTR retrotransposon *Fatima*

We performed a phylogenetic analysis of the *gypsy *LTR retrotransposons *Fatima *present in BAC_2383A24 and available in the public databases. All of the *Fatima *elements contained in the TREP database [42] fall into two groups, autonomous and nonautonomous. The "autonomous" variant presented TREP3189 by consensus nucleotide sequence and had two open reading frames corresponding to hypothetical proteins PTREP233 (polyprotein) and PTREP234. The "nonautonomous" variant presented TREP3198 by consensus nucleotide sequence and had open reading frames corresponding to hypothetical proteins PTREP231 (polyprotein) and PTREP232 (Figure 3). Using a BLASTP search [43] against the Pfam database [44], we demonstrated that PTREP231 contains *gag *and AP domains, while PTREP233 consists of RT, RH, INT, and AP domains and displays weak similarity to the *gag *domain. BLASTN alignments demonstrate that autonomous and nonautonomous elements have high similarity in the LTR region (91% identity over the entire length) and moderate similarity (65% over a 356-bp region) in the region corresponding to the aspartic proteinase domain. Sequence similarity between the remaining regions of autonomous and nonautonomous elements was undetectable. BAC_2383A24 contains representatives of both subfamilies; *Fatima*_2383A24- 2 and *Fatima*_2383A24-3 belong to the autonomous elements, and *Fatima*_2383A24-1p belongs to the nonautonomous elements. In the phylogenetic study, we analysed the autonomous and nonautonomous subfamilies separately because the internal regions of these elements are rather dissimilar in their sequences.

Using the consensus sequences TREP3189 (autonomous) and TREP3198 (nonautonomous) as reference sequences, we screened the NCBI nucleotide sequence database [45], including the high throughput genomic sequences division (HTGS) (for which sequencing is in progress) in the case of TREP3189. The genomic sequences belonging to *T. aestivum*, *T. durum*, *T. urartu*, *T. monococcum*, and *Ae. tauschii *showed significant BLAST hits (>75% identity over a region of >500 bp) to the reference sequences. The data on the analysed *Fatima *elements are consolidated in Additional File 2. From the HTG Sequences, we took only those ascribed to one of the common wheat genomes or genomes of its diploid relatives.

**Figure 4:**
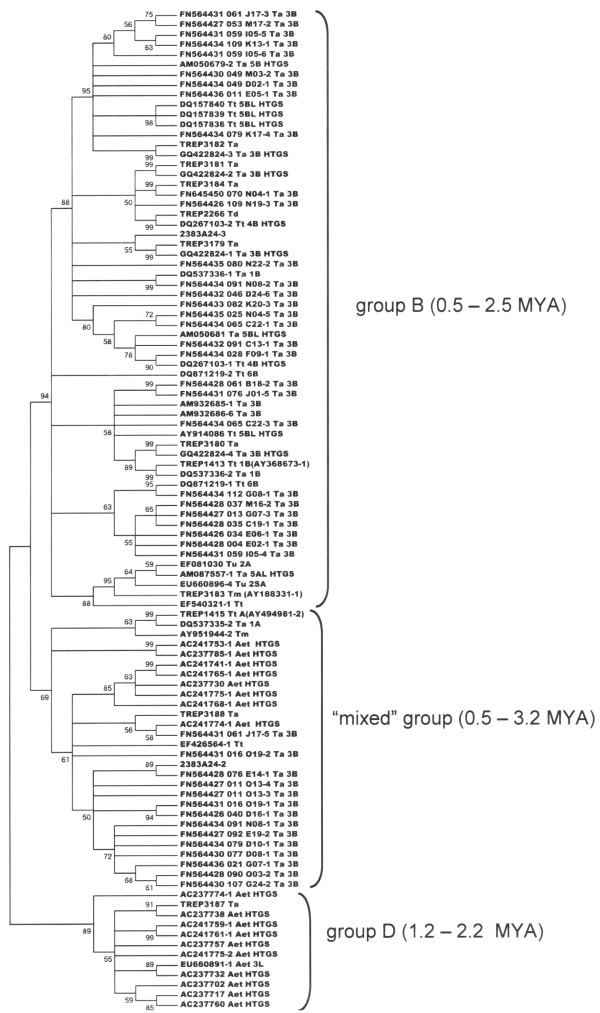
**The neighbor-joining phylogenetic tree of autonomous *Fatima *elements originating from different *Triticeae *genomes**. The phylogenetic tree was constructed using a CLUSTALW multiple alignment for the *Fatima *nucleotide sequences coding for RNase H. Bootstrap support over 50% is shown for the corresponding branches. Designations in sequence names: Ta, *T. aestivum; *Td, *T. durum; *Tt, *T. turgidum; *Tu, *T. urartu; *Tm, *T. monococcum; *and Aet, *Ae. tauschii*. Insertion timing for *Fatima *elements is parenthesised. The group designated as B predominantly contains the elements belonging to the B genome; and D, the elements belonging to the D genome. The "mixed" group contains the *Fatima *elements from different *Triticeae *genomes.

The regions homologous to the coding reference sequences were used in ClustalW multiple alignments [46] (see Methods). Multiple alignments were constructed individually for each conserved coding domain (AP, RT, RH, and INT for autonomous elements and GAG for nonautonomous elements). In total, we extracted 116 autonomous and 165 nonautonomous *Fatima *sequences from the public databases. We attributed *Fatima *sequences to particular genomes of allopolyploid wheat (where such data were available), as shown in Additional File 2 and Figure 4 (for autonomous elements). The insertion timing was estimated for each *Fatima *copy containing both LTR sequences (see Methods and Additional File 2).

For autonomous *Fatima *elements, we constructed the phylogenetic trees based on the nucleotide sequences coding for the conserved AP, RT, RH, or INT domains. All of the constructed phylogenetic trees for the autonomous elements had very similar topologies. The phylogenetic tree for the RH sequences (Figure 4) is shown as an example. In general, three main groups form the distinct branches on the trees. We designated the most abundant group as B-genome specific (or B-group) because it contains practically all of the *Fatima *elements from the B-genome chromosomes, except a subgroup of 5 elements from the A-genome. The element *Fatima*_2383A24-3, containing B-genome specific clone 2383A24/15, also falls into B-group. The insertion timing range for the elements of this branch is 0.5-2.5 MYA. The members of this group cluster separately from the elements originating from the elements of *Ae. tauschii *(D-genome specific group). The insertion timing for the elements of the D-genome specific group was determined for annotated sequences (1.2-2.2 MYA), as this group almost exclusively contains the elements found in unannotated HTG sequences. The group, referred to as a mixed group, forms a distinct cluster of the A-, B-, and D-genome specific subgroups (0.5-3.2 MYA). *Fatima*_2383A24-2 is a member of the B-genome specific subgroup.

Phylogenetic analysis of the nonautonomous group did not show any genome-specific clustering (data not shown). The insertion timing for the nonautonomous elements varies from 0.5 to 2.9 MYA; thus, the nonautonomous elements amplified approximately at the same time as the autonomous elements (see Additional File 2).

## Discussion

### BAC_2383A24 probes provide a means of identifying the chromosomes of the allopolyploid wheat B-genome and *Ae. speltoides *with various backgrounds

The genus *Triticum *comprises diploid, tetraploid, and hexaploid species with a basic chromosome number multiple of seven (*x *= 7). One of the approaches to studying plant genomes with a common origin is *in situ *hybridisation using total genomic DNA as a probe, or GISH [47-49]. This method makes it possible to concurrently estimate the similarity of repeated sequences and chromosomal rearrangement (translocations) during evolution, detect interspecific and even intraspecific (interpopulation) polymorphisms, and identify foreign chromosomes and their segments in a particular genetic background. The difficulties encountered in discriminating between the genomes of allopolyploid species using GISH result from the following two issues:

(1) "fitting" of the genomes that composed the allopolyploid nucleus during the evolution of the allopolyploid species, which involved homogenization of repeated sequences and redistribution of mobile elements, and

(2) the genomes of diploid progenitors for an allopolyploid species are rather close to one another, with few divergent representations of repeated sequences.

GISH analysis of *Nicotiana *allopolyploids provided direct evidence for a decrease in the divergence between the parental genomes during the evolution via exchange and homogenisation of repeats [49]. It has been demonstrated that GISH is able to distinguish between the constituent genomes in the first generation of synthetic *Nicotiana *allopolyploids. The parental genomes of an allopolyploid formed as long ago as 0.2 MYA are similarly easy to distinguish; however, the parental genomes in this case display numerous translocations. The efficiency of GISH considerably decreases when analysing the *Nicotiana *allopolyploids formed about 1 MYA, thereby suggesting a considerable exchange of repeats between parental chromosome sets [49].

It has been suggested that close affinities among the diploid donor species *T. urartu*, *Ae. speltoides*, and *Ae. tauschii *interfere with a GISH-based discrimination between different genomes in hexaploid wheat [16]. Our results from simultaneous *in situ *hybridisation of BAC_2383A24 and *Ae. tauschii *genomic DNA to the slide containing both *Ae. speltoides *and *Ae. tauschii *cells demonstrate a clear discrimination between the chromosomes of these diploid species (Figure 1E). The differences between the genomes are also detectable when hybridising BAC_2383A24 with the metaphase chromosomes of *Ae. speltoides *and *T. urartu *(Figure 1D). Similar to *Nicotiana *allopolyploids, the efficiency of genome discrimination decreases in the cases of tetraploid and hexaploid wheat, likely due to increased cross-hybridisation of the BAC_2383A24 (B-genome) repeats and *Ae. tauschii *genomic DNA with chromosomes from homoeologous genomes. The formation of Emmer wheat dates back to 0.5 MYA; judging from the dating for rearrangements in *Nicotiana *allopolyploids, this is a sufficient time period for considerable rearrangements in the TE fraction between the parental chromosome sets.

Simultaneous hybridisation using BAC_2383A24 (B-genome) and the probes that provide for identification of common wheat chromosomes demonstrated that BAC_2383A24 is able to detect translocations involving the B-genome that occurred during the evolution of the allopolyploid emmer wheat (Figure 1A).

*In situ *hybridisation demonstrated a dispersed localisation for the majority of BAC clones on wheat chromosomes (as in the case of BAC_2383A24), which can be explained by the fact that BAC clones contain various TEs with disperse genomic localisations [50]. Analysis of the complete BAC_2383A24 nucleotide sequence (totaling 113 605 bp) demonstrated that mobile elements constitute 55.6% of the sequence, the most abundant being LTR retrotransposons (51.6% of the clone). Most predominant among the retrotransposons is the *gypsy *LTR retrotransposon family *Fatima*, constituting up to 47.2% of all LTR retrotransposons. The results of BAC subcloning and subsequent *in situ *hybridisation of subclone 2383A24/15 (Figure 1B) suggest that the *Fatima *family elements significantly contribute to the BAC_2383A24 B-genome specific FISH pattern.

Several reasons can explain a genome-specific BAC-FISH pattern, namely, (1) the presence of specific TE families and (2) differences in proliferation of the same TEs in different genomes.

### Estimating the contribution of *Fatima *to the divergence and differentiation of the B-genome

In assessing TE contribution to the differentiation of the genomes in hexaploid wheat, it is reasonable to turn to earlier works estimating the content of repeated DNA sequences and heterochromatin in wheat and their progenitors. In particular, all three genomes that form hexaploid wheat considerably differ in the content of their repeated DNA fraction involved in formation of the heterochromatic chromosomal regions. C-banding demonstrates that the B-genome is the richest in heterochromatin, the A-genome is the poorest, and the D-genome occupies the intermediate position [51]. A high heterochromatin content in the B-genome correlates with the size of this genome, which amounts to 7 pg and exceeds the sizes of the diploid wheat species [11]. It was later demonstrated that the satellite GAA was one of the main components of the B-genome heterochromatin, and the families of tandem repeats pSc119.2 and pAs1 were detected. Notably, their localisation partially coincides with the localisation of heterochromatic blocks in common wheat [36]. The 120-bp tandem repeat pSc119.2 predominantly clusters on the B-genome chromosomes and individual D-genome chromosomes, whereas the pAs1 (or *Afa *family) clusters on the D-genome chromosomes and individual A- and B-genome chromosomes. The distinct localisation of these repeats in certain chromosomal regions allows their use as probes for chromosome identification [36]. As has been demonstrated, the diploid progenitors of the corresponding polyploid wheat genomes also differ in the content of these repeats.

In 1980, Flavell studied the repeated sequences of *T. monococcum*, *Ae. speltoides*, and *Ae. tauschii *and demonstrated that each species contains a certain fraction of species-specific repeats. This fraction is the largest in *Ae. speltoides*, constituting 2% of the total genomic DNA. As for the diploid with the A-genome, the content of species-specific repeats is lower than in the species that donated the B- and D-genomes. Part of the *Ae. speltoides *species-specific repeats can be explained by the presence of the high copy number subtelomeric tandem repeat family Spelt1 [26]. Evidently, the genome-specific variants of the pSc119.2 family can contribute to this fraction.

Thus, previous results suggest that the B-genome differs from the other genomes of hexaploid wheat with a higher content of distinct tandem repeat families, some of which are B-genome specific.

TEs also impact B-genome specificity. The advent of wheat BAC clones and their sequencing makes it possible to consider in more detail the differentiation of the parental genomes in hexaploid wheat and the involvement of repeated DNA sequences in this process, namely TEs, as their most represented portion. In a recent study analysing TE representation in 1.98 Mb of B genomic sequences and 3.63 Mb of A genomic sequences, we showed that TEs of the *Gypsy *superfamily have proliferated more in the B-genome, whereas those of the *Copia *superfamily have proliferated more in the A-genome [6]. In addition, this comparison demonstrated that the *Fatima *family is more abundant in the B-genome among the *gypsy*-like elements and that the *Angela *family is more abundant in the A-genome among the *copia*-like elements [6]. When analysing BAC_2383A24, which we localised to the 3B chromosome, we also demonstrated that *gypsy *elements are more abundant than *copia *elements and that *Fatima *constitutes 85.8% of all *gypsy *elements annotated in this clone (Table 1, Figure 2). A comparison of 11 Mb of random BAC end sequences from the B-genome with 2.9 Mb of random sequences from the D-genome of *Ae. tauschii *demonstrated that the *athila*-like *Sabrina *together with *Fatima *elements, are the most abundant TE families in the D-genome [7].

A study of the distribution of *gypsy*-like *Fatima *elements in the common wheat genome by *in situ *hybridisation with the probes 2383A24/15 (a *Fatima *element) and BAC_2383A24 (where *Fatima *elements constitute 23.9% of its length) has revealed a B-genome specific FISH pattern (Figure 1). Most likely, the observed hybridisation patterns of *Fatima *elements with the common wheat chromosomes is determined by higher proliferation of *Fatima *sequences in the B-genome and/or the presence of the B-genome specific variants of *Fatima *sequences.

Analysis of the wheat DNA sequences available in databases demonstrated that *Fatima *elements are present in all the three genomes (A, B, and D) of common wheat. Phylogenetic analysis confirms that the autonomous *Fatima *elements fall into B-genome-, D-genome- and A-genome-specific groups and subgroups (Figure 4). The *Fatima_*2383A24-3 element (2383A24/15) belongs to the B-genome-specific group. *Fatima *2383A24-2 belongs to the B-genome subgroup, which together with A-genome and D-genome subgroups form a mixed group. Insertion of the *Fatima *elements that form the B-genome-, A-genome- and D-genome-specific groups and subgroups took place in the time interval 0.5-3.2 MYA (Figure 4). This time corresponds to the period between formation of the diploid species and their hybridisation, which led to the wild Emmer tetraploid wheat *T. dicoccoides *[20,21,30]. The insertion time of *Fatima*_2383A24-3, predominantly localised to the B-genome (Figure 1), is 1.6 MYA, which matches the proliferation of the B-genome-specific groups in the diploid progenitor.

Therefore, B-genome specificity of the *gypsy*-like *Fatima *as determined by FISH is, to a great degree, explained by the appearance of a genome-specific element within this family from *Ae. speltoides*, the diploid progenitor of the B-genome. Likely, its proliferation mainly occurred in this diploid species before it entered into allopolyploidy, as suggested by both the BAC FISH data (Figure 1) and phylogenetic analysis (Figure 4). Most likely, this scenario of emergence and proliferation of the genome-specific variants of retroelements in the diploid species is characteristic of the evolution of all three genomes in hexaploid wheat. The fact that over 80% of the TEs in the A- and B-genomes proliferated before the formation of allopolyploid wheat also confirms this hypothesis [6]. Note that the B-genome-specific elements are not only present in the Ty3-*gypsy Fatima *family. In particular, *in situ *hybridisation of the RT fragment from *Ae. speltoides *Ty1-*copia *retroelements (RT probe) to the *T. diccocoides *chromosomes distinguished between the A- and B-genome chromosomes. The RT probe displayed the most intensive hybridisation to B-genome chromosomes [27].

Note also the observed decrease in the efficiency of BAC FISH identification of the B-genome in allopolyploid wheat (Figure 1) compared with the diploid progenitors. This suggests that the transpositions of the *gypsy *LTR retrotransposon family *Fatima *and possibly other genome-specific TEs occurred after the formation of allopolyploids.

## Conclusions

In this work, we performed a detailed analysis of the *T. aestivum *clone BAC-2383A24 and the Ty3-*gypsy *group LTR retrotransposons *Fatima*. BAC_2383A24, marked by a subtelomeric Spelt1 repeat, was localized in a distal region on the short arm of 3B chromosome using ISBP marker and the data on a synteny of wheat and rice chromosomes. Interestingly, characteristic of BAC_2383A24 is a higher gene density (one gene per 23 kb) and a lower TE content (55.6%) relative to the mean values currently determined for the wheat genome, which is in general characteristic of the distal region of the short arm of 3B chromosome [8]. Further physical mapping and sequencing of individual wheat chromosomes will clarify whether a high gene density and a lower TE content are specific features of this chromosome region only or this is also characteristic of other distal chromosome regions.

The *gypsy *LTR retrotransposon *Fatima *is the most abundant in BAC_2383A24 and, similar to the overall clone, is predominantly localized to the B-genome chromosomes of polyploid and diploid wheat species. Given the data from FISH and the phylogenetic analysis of the *Fatima *elements taken from public databases, we concluded that the observed hybridisation pattern of *Fatima *elements to the common wheat chromosomes was due to higher proliferation of *Fatima *sequences in the B-genome and the presence of B-genome specific variants of *Fatima *sequences. According to our estimates, proliferation of B-genome specific variants of elements took place in the time interval 0.5-2.5 MYA, which corresponds to the time period between when the diploid B-genome progenitor species *Ae. speltoides *formed and before the hybridisation event that led to formation of the wild Emmer tetraploid wheat *T. dicoccoides*. Most likely, this scenario of emergence and proliferation of genome-specific variants of retroelements, mainly in the diploid species, is characteristic of the evolution of all three genomes in hexaploid wheat.

## Methods

The selection of BAC_2383A24 from the genomic BAC library of *T. aestivum *cv. Renan was described by [35].

### Plant material

The species *T. urartu *Tum. (genomic formula, A^u^A^u^) TMU06, *Ae. speltoides *Tausch (genomic formula, SS) TS01, and *Ae. tauschii *Coss. (genomic formula, DD) TQ27 were kindly provided by M. Feldman, the Weizmann Institute of Science, Israel. *T. durum *Desf. (genomic formula, BBAA) cv. Langdon, *T. aestivum *L. (genomic formula, BBAADD) cvs. Chinese Spring, and Renan and Saratovskaya 29 were maintained in the Institute of Cytology and Genetics, Novosibirsk, Russia.

### PCR analysis

The following specific primer pairs designed for the junctions of LTR retroelements were used: *Barbara*_2383A24-1p/*Fatima*_2383A24-2 (BarbL, 5'-ccaga-taccc-attca-ccaac-3' and BarbR, 5'-ccgag-gagca-caacc-ttac-3'). The PCR mixture contained 100 ng of *Triticum *or *Aegilops *genomic DNA, 1 × PCR buffer (67 mM Tris-HCl pH 8.8, 18 mM (NH_4_)_2_SO_4_, 1.7 mM MgCl_2_, and 0.01% Tween 20), 0.25 mM of each dNTP, 0.5 μM of each primer, 1 U of *Taq *polymerase, and deionized water to a final volume of 25 μl. PCR was performed in an Eppendorf Mastercycler according to the following mode: 35 cycles of 1 min at 94°C, 1 min at 60°C, and 2 min at 72°C, followed by a final stage of 15 min at 72°C. PCR products were separated by electrophoresis in a 1% agarose gel.

### Fluorescence *in situ *hybridisation (FISH)

Fluorescent *in situ *hybridisation experiments were done as described in detail by [26]. Probes were labeled with biotin and digoxigenin and then detected with avidin-FITC (green) and an anti-digoxigenin-rhodamine Fab fragment (red). BAC_2383A24 was hybridized to a set of slides containing the metaphase chromosomes for the polyploid species (1) *T. aestivum *and (2) *T. durum *as well as two diploid species simultaneously, namely, (3) *T. urartu *and *Ae. speltoides*, (4) *Ae. tauschii *and *Ae. speltoides*. Subclone 2383A24/15 was hybridized to *T. aestivum*. To distinguish between the B- and D-genome chromosomes, we co-hybridized the probes under study with clones pSc119.2 and pAs1, respectively [52]. Total *Ae. tauschii *DNA was used as a probe for the D-genome chromosomes.

### BAC subcloning and colony hybridisation

To extract DNA fragments from BAC_2383A24 that hybridize specifically to the B-genome, we performed BAC subcloning and subsequent hybridisation with α-^32^P-labeled *Ae. speltoides *genomic DNA. Initially, we obtained a set of 250 *Sau*3AI fragments ranging in size from 100 to 1000 bp cloned in the *Bam*HI-digested pUC18 (Promega, USA). The colonies were then transferred to a Hybond N+ membrane [53] and hybridized with the probe labeled by the random hexamer method using α-^32^P-dATP (Amersham Pharmacia Biotech, UK) and a Klenow fragment [54]. The hybridisation mixture also contained competitive *T. urartu *and *Ae. tauschii *genomic DNA in the same quantities as the *Ae. speltoides *genomic probe (100 ng each per 20 ml of hybridisation mixture). Filters were first moistened by floating on 2 × SSC. Prehybridisation was performed at 65°C for 4 h in 6 × SSC, 5 × Denhardt's solution, 0.5% SDS, and denatured salmon sperm DNA (100 μg/ml). Hybridisation was performed in the same solution with denatured, labeled probe and competitive DNA for 16 h. After hybridisation, filters were washed at room temperature for 15 min in each of the following solutions: 2 × SSC, 0.1% SDS; 0.5 × SSC, 0.1% SDS; and 0.1 × SSC, 0.1% SDS. The membranes were exposed with Kodak X-ray film for 3 days at -70°C.

### Analysis of the BAC_2383A24 nucleotide sequence

The sequences were determined using the random shotgun method at the National Center of Sequencing (Evry, France) as described by Chantret et al. [28]. Briefly, the BAC clone was sequenced using Sanger technology at 20 X final coverage. After sequence assembly, finishing of gaps were performed by sequencing of PCR products with primers designed on sequencing flanking the gaps, until one single contig was built. Lastly sequence assembly was verified by long-range (10 kb) PCR covering the BAC clone. The resulting sequence of 113 605 bp was annotated according to Charles et al. [6] and the Guidelines for Annotating Wheat Genomic Sequences from International Wheat Genome Sequencing Consortium [http://www.wheatgenome.org/Tools-and-Resources/Bioinformatics-Board/Annotation-Guidelines]. The DNA sequences that were not assigned to transposable elements or genes were regarded as unassigned DNA. The BAC_2383A24 nucleotide sequence determined in this work was deposited in GenBank under the accession number [GenBank: GU817319].

### Database screening for *Fatima *elements

For phylogenetic analysis of the *Fatima *family elements, we compiled a dataset containing the autonomous and nonautonomous LTR retrotransposon *Fatima *sequences currently available in the TREP [42] and NCBI Nucleotide Sequence Databases [45] (Additional File 2). The *Fatima *elements were searched for using the BLASTn algorithm [43] and the consensus sequences TREP3189 and TREP3198 as reference sequences.

### Phylogenetic analysis of *Fatima *sequences coding for conserved domains

Phylogenetic analysis was performed separately for autonomous and nonautonomous elements. In the case of autonomous elements, phylogenetic analysis was based on the nucleotide sequences corresponding to conserved functional domains AP, RT, RH, and INT. The nucleotide sequences coding for individual domains were determined using BLASTX-2. The consensus amino acid sequences of the functional domains AP, RT, RH, and INT for autonomous *Fatima *elements were determined by a BLASTP comparison hypothetical polyprotein PTREP233 consensus sequence and the sequences of functional domains and proteins in the Pfam and NCBI databases. In the case of nonautonomous elements, phylogenetic analysis was performed using the sequences corresponding to the functional domains GAG and AP. The nucleotide sequences encoding these functional domains were determined similar to the domains for autonomous elements; the consensus hypothetical protein PTREP231 was used for obtaining the consensus amino acid sequences for the GAG and AP domains. The nucleotide sequences of analyzed elements corresponding to the same functional domains were multiply aligned using the ClustalW program with the MEGA4 software package [46,55]. Phylogenetic trees were constructed by the neighbor-joining method with the help of MEGA4 software and a maximum likelihood model with 500 bootstrap replicates and pairwise nucleotide deletion options.

### Dating the LTR retrotransposon insertion

For dating the insertion events of the autonomous and nonautonomous *Fatima *elements, we analyzed the nucleotide divergence rate between two LTRs in the case when both LTRs were present in the elements' structure. To determine the LTR boundaries, each element was compared with itself using Blast2seq [http://www.ncbi.nlm.nih.gov/blast/bl2seq/wblast2.cgi]. In addition, the presence of the characteristic motifs, 5'-TG-3' and 5'-CA-3', at the beginning and end of each LTR, respectively, was taken into account. Each pair of LTRs was aligned using the ClustalW algorithm with the MEGA4 program. The degree of divergence (with standard error, SE) was calculated using the Kimura two-parameter method [56] and complete deletion option. To convert this term into the insertion date, we used the following equation: *T *= *D*/2*r*, where *T *is the time elapsed since the insertion; *D*, the estimated LTR divergence; and *r*, the substitution rate per site per year [57]. We applied a substitution rate of 1.3 × 10^-8 ^mutations per site per year for the plant LTR retrotransposons [58].

## Abbreviations

(BAC): Bacterial artificial chromosome; (TE): transposable element; (FISH): fluorescent *in situ *hybridization; (LTR): long terminal repeats; (RT): reverse transcriptase;

## Authors' contributions

EMS, ABS and EAS carried out the molecular genetic studies and data analysis; IGA performed FISH analysis. HB and CH carried out the BAC clone sequencing. EAS drafted and edited the manuscript. BC conducted the coordination of BAC analysis and the manuscript conception. All authors read and approved the final manuscript.

## Supplementary Material

Additional file 1 **The applied ISBP method for BAC_2383 localisation**. (A) The positions of BarbL and BarbR primers relative to the insertions of *Barbara*_2383A24-1p and *Fatima*_2383A24-2 retroelements in BAC_2383A24 clone. The studied region is marked by a dashed rectangle. (B) Electrophoretic analysis of the PCR products with specific BarbL and BarbR primers on the nullitetrasomic lines of *T. aestivum *cv. Chinese Spring. The line N3BT3D lacks a specific PCR fragment. Click here for file

Additional file 2 **The analysed Fatima elements**. The list of Fatima elements used for phylogenetic analysis. The attribution of *Fatima *sequences to particular genomes of allopolyploid wheat (if such data are available) is shown, and the estimation of insertion time based on LTR divergence is included. Click here for file
