# Supplementary material for: The impact of Ty3-gypsy group LTR retrotransposons Fatima on B-genome specificity of polyploid wheats
Source: BMC Plant Biol. 2011 Jun 3;11:99. doi: 10.1186/1471-2229-11-99 (PMC3129301; doi:10.1186/1471-2229-11-99)

**Additional file 1**

**Applied ISBP method for BAC_2383A24 localization.**

**A.** The positions of BarbL and BarbR primers relative to the insertions of *Barbara*_2383A24-1p and *Fatima*_2383A24-2 retroelements in BAC_2383A24 clone. The studied region is marked by dashed rectangle.

**B.** Electrophoretic analysis of the PCR products with specific BarbL and BarbR primers on the nullitetrasomic lines of *T. aestivum* cv. Chinese Spring. The line N3BT3D lacks specific PCR fragment.

**A.**

**
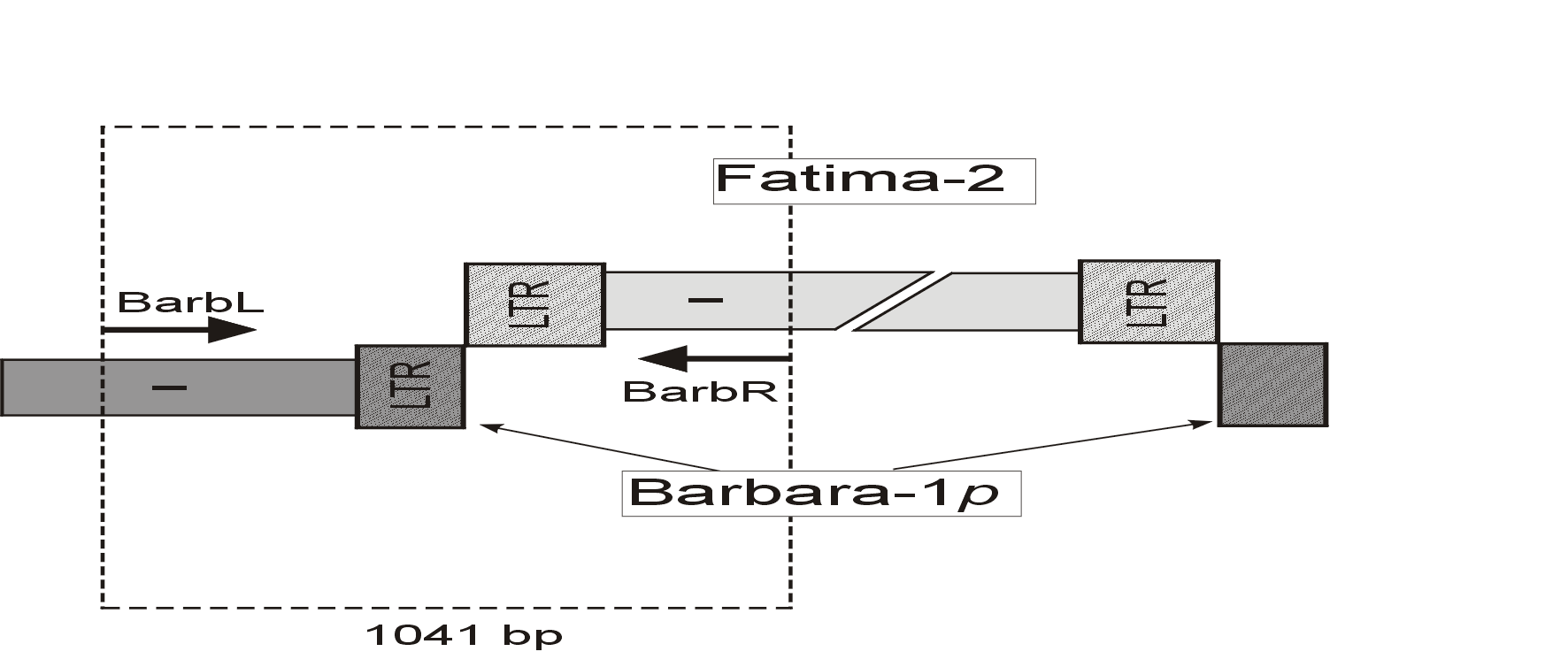
**

**B.**


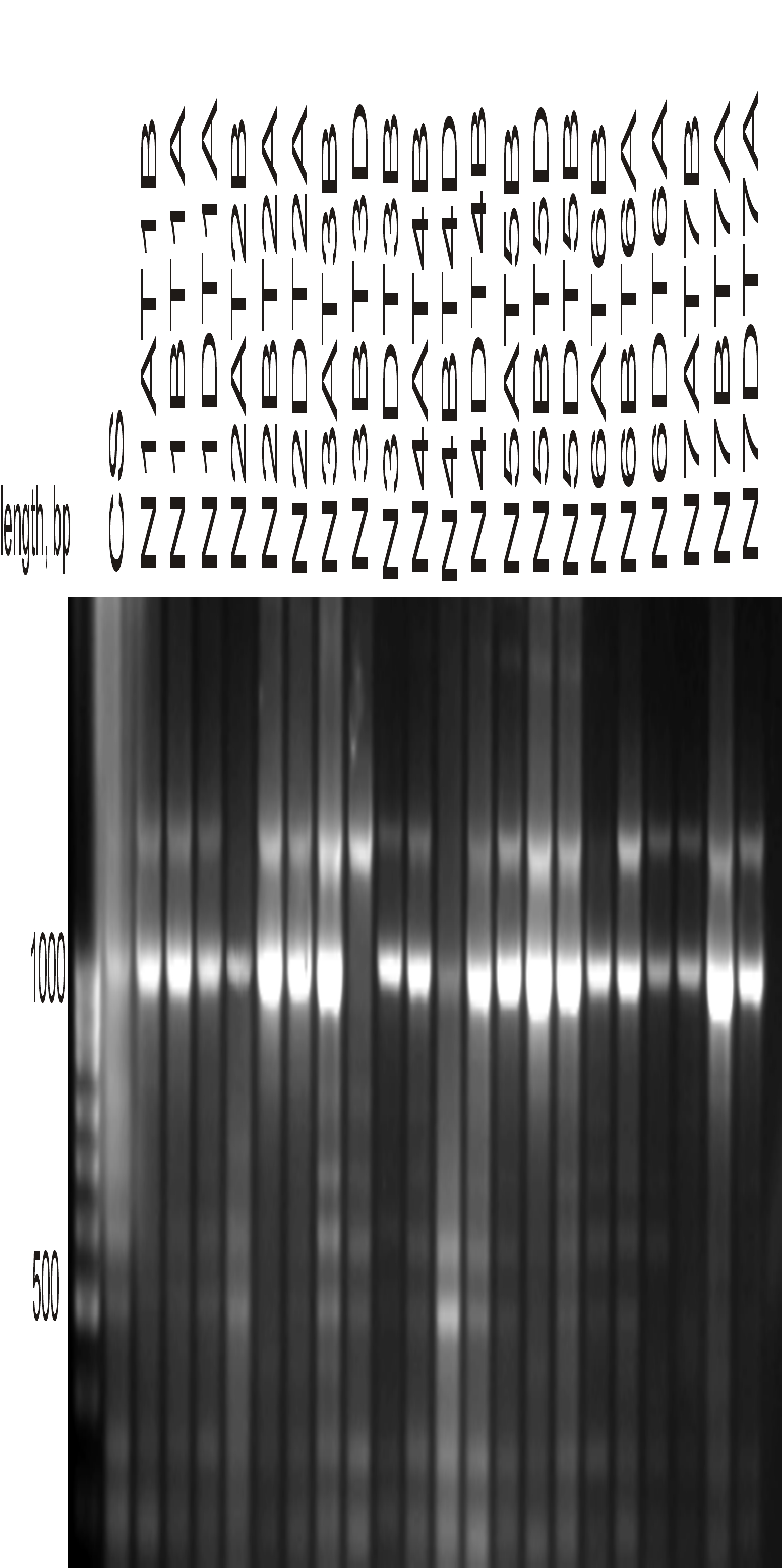

Supplement: Additional file 1 — The applied ISBP method for BAC_2383 localisation. (A) The positions of BarbL and BarbR primers relative to the insertions of Barbara_2383A24-1p and Fatima_2383A24-2 retroelements in BAC_2383A24 clone. The studied region is marked by a dashed rectangle. (B) Electrophoretic analysis of the PCR products with specific BarbL and BarbR primers on the nullitetrasomic lines of T. aestivum cv. Chinese Spring. The line N3BT3D lacks a specific PCR fragment. [file 1471-2229-11-99-S1.DOC]
