# Supplementary material for: The impact of Ty3-gypsy group LTR retrotransposons Fatima on B-genome specificity of polyploid wheats
Source: BMC Plant Biol. 2011 Jun 3;11:99. doi: 10.1186/1471-2229-11-99 (PMC3129301; doi:10.1186/1471-2229-11-99)
Supplement: Additional file 2 — The analysed Fatima elements. The list of Fatima elements used for phylogenetic analysis. The attribution of Fatima sequences to particular genomes of allopolyploid wheat (if such data are available) is shown, and the estimation of insertion time based on LTR divergence is included. [file 1471-2229-11-99-S2.DOC]

**Additional file 2.**

**The analyzed Fatima elements**

| **Autonomous elements** | | | | | | | |
| --- | --- | --- | --- | --- | --- | --- | --- |
| № | **Accession** | **Sequence is belong to species (genome or chromosome)** | **PTREP**  **233** | **PTREP**  **234** | **Number of LTR** | **LTR Divergence (Insertion time in MYA)** | **Standard error (time in MYA)** |
|  | 2383А24-2 | *T. aestivum* (3В) | + | + | 2 | 0.061 (2.35) | 0.012 (0.46) |
|  | 2383А24-3 | *T. aestivum* (3В) | + | + | 2 | 0.042 (1.62) | 0.009 (0.35) |
|  | TREP2266 (103H9-1) | *T. durum* | + | + | 2 | 0.035 (1.30) | 0.009 (0.35) |
|  | TREP3179 | *T. aestivum* | + | + | 2 | 0.035 (1.35) | 0.009 (0.35) |
|  | TREP3180 | *T. aestivum* | + | + | 2 | 0.039 (1.50) | 0.008 (0.31) |
|  | TREP3181 | *T. aestivum* | + | + | 2 | 0.022 (0.85) | 0.007 (0.27) |
|  | TREP3182 | *T. aestivum* | + | + | 2 | 0.024 (0.92) | 0.007 (0.27) |
|  | TREP3183 (AY188331-1)( 231A16-1) | *T. monococcum* (5AL) | + | + | 2 | 0.044 (1.70) | 0.010 (0.38) |
|  | TREP3184 | *T. aestivum* | + | + | 2 | 0.024 (0.92) | 0.007 (0.27) |
|  | TREP3185 | *T. aestivum* | + | + | 2 | 0.067 (2.58) | 0.012 (0.46) |
|  | TREP3187 | *T. aestivum* | + | + | 2 | 0.058 (2.23) | 0.011 (0.42) |
|  | TREP3188 | *T. aestivum* | + | + | 2 | 0.060 (2.31) | 0.011 (0.42) |
|  | TREP1413 (AY368673-1) (1126E20-1) | *T. turgidum* (1B) | + | + | 2 | 0.039 (1.50) | 0.009 (0.35) |
|  | TREP1415 (AY494981-2) (634M12-2) | *T. turgidum* (A) | + | + | 2 | 0.042 (1.62) | 0.010 (0.38) |
|  | DQ537335-2 | *T. aestivum* (1A) | + | + | 2 | 0.049 (1.88) | 0.011 (0.42) |
|  | DQ537336-1 | *T. aestivum* (1B) | + | + | 2 | 0.017 (0.65) | 0.006 (0.23) |
|  | DQ537336-2 | *T. aestivum* (1B) | + | + | 2 | 0.033 (1.27) | 0.008 (0.31) |
|  | DQ871219-1 (916O17-1) | *T. turgidum* (6B) | + | + | 2 | 0.037 (1.42) | 0.009 (0.35) |
|  | DQ871219-2(409D13-1) | *T. turgidum* (6B) | + | + | 2 | 0.039 (1.50) | 0.009 (0.35) |
|  | AM932686-6 (TA3B95G2-6) | *T. aestivum* (3B) | + | + | 2 | 0.051 (1.96) | 0.010 (0.38) |
|  | EF540321-1 (326E2-1) | *T. turgidum* | + | + | 2 | 0.040 (1.54) | 0.009 (0.35) |
|  | AY951944-2 (21C6-2) | *T. monococcum* | + | + | 2 | 0.044 (1.69) | 0.010 (0.38) |
|  | EU660896-4 | *T. urartu* (2AS) | + | + | 2 | 0.019 (0.73) | 0.005 (0.19) |
|  | FN645450 (3B_070_N04-1) | *T. aestivum* (3B) | + | + | 2 | 0.024 (0.92) | 0.007 (0.27) |
|  | FN564426(3B_109_N19-3) | *T. aestivum* (3B) | + | + | 2 | 0.024 (0.92) | 0.007 (0.27) |
|  | FN564426 (3B_034_E06-1) | *T. aestivum* (3B) | + | + | 2 | 0.041 (1.58) | 0.009 (0.35) |
|  | FN564426 (3B_040_D16-1) | *T. aestivum* (3B) | + | + | 2 | 0.046 (1.77) | 0.010 (0.38) |
|  | FN564436 (3B_011_E05-1) | *T. aestivum* (3B) | + | + | 2 | 0.028 (1.08) | 0.008 (0.31) |
|  | FN564436 (3B_021_G07-1) | *T. aestivum* (3B) | + | + | 2 | 0.045 (1.73) | 0.009 (0.35) |
|  | FN564434 (3B_079_K17-4) | *T. aestivum* (3B) | + | + | 2 | 0.028 (1.08) | 0.008 (0.31) |
|  | FN564434 (3B_091_N08-2) | *T. aestivum* (3B) | + | + | 2 | 0.017 (0.65) | 0.006 (0.23) |
|  | FN564434 (3B_112_G08-1) | *T. aestivum* (3B) | + | + | 2 | 0.028 (1.08) | 0.007 (0.27) |
|  | FN564434 (3B_028_F09-1) | *T. aestivum* (3B) | + | + | 2 | 0.020 (0.77) | 0.006 (0.23) |
|  | FN564434 (3B_109_K13-1) | *T. aestivum* (3B) | + | + | 2 | 0.013 (0.5) | 0.005 (0.19) |
|  | FN564434 (3B_065_C22-1) | *T. aestivum* (3B) | + | + | 2 | 0.012 (0.46) | 0.005 (0.19) |
|  | FN564434 (3B_065_C22-3) | *T. aestivum* (3B) | + | + | 2 | 0.042 (1.62) | 0.010 (0.38) |
|  | FN564434 (3B_091_N08-1) | *T. aestivum* (3B) | + | – | 2 | 0.042 (1.62) | 0.010 (0.38) |
|  | FN564434 (3B_079_D10-1) | *T. aestivum* (3B) | + | + | 2 | 0.052 (2.00) | 0.011 (0.42) |
|  | FN564430 (3B_049_M03-2) | *T. aestivum* (3B) | + | + | 2 | 0.030 (1.15) | 0.010 (0.38) |
|  | FN564430 (3B_107_G24-2) | *T. aestivum* (3B) | + | + | 2 | 0.050 (1.92) | 0.011 (0.42) |
|  | FN564430 (3B_066_H03-1) | *T. aestivum* (3B) | + | + | 2 | 0.056 (2.15) | 0.011 (0.42) |
|  | FN564430 (3B_077_D08-1) | *T. aestivum* (3B) | + | + | 2 | 0.058 (2.23) | 0.012 (0.46) |
|  | FN564427 (3B_053_M17-2) | *T. aestivum* (3B) | + | + | 2 | 0.028 (1.08) | 0.008 (0.31) |
|  | FN564427 (3B_013_G07-3) | *T. aestivum* (3B) | + | + | 2 | 0.028 (1.08) | 0.008 (0.31) |
|  | FN564427 (3B_011_O13-3) | *T. aestivum* (3B) | + | + | 2 | 0.063 (2.42) | 0.012 (0.46) |
|  | FN564427 (3B_011_O13-4) | *T. aestivum* (3B) | + | + | 2 | 0.072 (2.77) | 0.013 (0.50) |
|  | FN564427 (3B_092_E19-2) | *T. aestivum* (3B) | + | + | 2 | 0.028 (1.08) | 0.008 (0.31) |
|  | FN564427 (3B_078_G10-2) | *T. aestivum* (3B) | + | + | 2 | 0.077 (2.96) | 0.014 (0.54) |
|  | FN564427 (3B_078_G10-1) | *T. aestivum* (3B) | + | + | 2 | 0.044 (1.69) | 0.010 (0.38) |
|  | FN564431 (3B_059_I05-6) | *T. aestivum* (3B) | + | + | 2 | 0.064 (2.46) | 0.012 (0.46) |
|  | FN564431 (3B_059_I05-5) | *T. aestivum* (3B) | + | + | 2 | 0.033 (1.27) | 0.009 (0.35) |
|  | FN564431 (3B_061_J17-3) | *T. aestivum* (3B) | + | + | 2 | 0.028 (1.08) | 0.008 (0.31) |
|  | FN564431 (3B_076_J01-5) | *T. aestivum* (3B) | + | + | 2 | 0.037 (1.42) | 0.009 (0.35) |
|  | FN564431 (3B_016_O19-1) | *T. aestivum* (3B) | + | + | 2 | 0.062 (2.38) | 0.012 (0.46) |
|  | FN564431 (3B_016_O19-2) | *T. aestivum* (3B) | + | + | 2 | 0.074 (2.85) | 0.013 (0.50) |
|  | FN564431 (3B_059_I05-4) | *T. aestivum* (3B) | + | + | 2 | 0.047 (1.81) | 0.010 (0.38) |
|  | FN564431 (3B_061_J17-5) | *T. aestivum* (3B) | + | + | 2 | 0.060 (2.31) | 0.012 (0.46) |
|  | FN564435 (3B_080_N22-2) | *T. aestivum* (3B) | + | + | 2 | 0.033 (1.27) | 0.009 (0.35) |
|  | FN564435 (3B_025_N04-5) | *T. aestivum* (3B) | + | + | 2 | 0.050 (1.92) | 0.014 (0.54) |
|  | FN564428 (3B_004_E02-1) | *T. aestivum* (3B) | + | + | 2 | 0.069 (2.65) | 0.013 (0.50) |
|  | FN564428 (3B_037_M16-2) | *T. aestivum* (3B) | + | + | 2 | 0.039 (1.5) | 0.009 (0.35) |
|  | FN564428 (3B_035_C19-1) | *T. aestivum* (3B) | + | + | 2 | 0.029 (1.12) | 0.008 (0.31) |
|  | FN564428 (3B_061_B18-2) | *T. aestivum* (3B) | + | + | 2 | 0.028 (1.08) | 0.008 (0.31) |
|  | FN564428 (3B_090_O03-2) | *T. aestivum* (3B) | + | + | 2 | 0.030 (1.15) | 0.008 (0.31) |
|  | FN564428 (3B_076_E14-1) | *T. aestivum* (3B) | + | + | 2 | 0.082 (3.15) | 0.014 (0.54) |
|  | FN564432 (3B_091_C13-1) | *T. aestivum* (3B) | + | + | 2 | 0.028 (1.08) | 0.008 (0.31) |
|  | FN564432 (3B_046_D24-6) | *T. aestivum* (3B) | + | + | 2 | 0.024 (0.92) | 0.007 (0.27) |
|  | AM932685-1 (TA3B95F5-1) | *T. aestivum* (3B) | + | + | 1 |  |  |
|  | EF081030-1 | *T. urartu* (2A) | + | + | 1 |  |  |
|  | FN564434 (3B_049_D02-1) | *T. aestivum* (3B) | + | + | 1+fr |  |  |
|  | FN564433 (3B_082_K20-3) | *T. aestivum* (3B) | + | + | - |  |  |
|  | FN645450 (3B_110_D05-1) | *T. aestivum* (3B) | + | – | 1 |  |  |
|  | EF426564-1 | *T. turgidum* | + | – | 1 |  |  |
|  | EU660891-1 | *Ae. tauschii* (3L) | + | – | 1 |  |  |
|  | TREP3186 (609E6-2) | *T. monococcum* | – | + | 1 |  |  |
|  | AM932683-1 (TA3B81B7-1) | *T. aestivum* (3B) | – | + | 1 |  |  |
| **Autonomous elements from High Throughput Genomic Sequences (sequencing in progress)** | | | | | | | |
|  | AC237702 | *Ae. tauschii* | + |  | 2 | 0.036 (1.38) | 0.009 (0.35) |
|  | AC237730 | *Ae. tauschii* | + |  | 2 | 0.013 (0.50) | 0.005 (0.19) |
|  | AC237732 | *Ae. tauschii* | + |  | 2 | 0.031 (1.19) | 0.008 (0.31) |
|  | AC237738 | *Ae. tauschii* | + |  | 2 | 0.049 (1.88) | 0.011 (0.42) |
|  | AC237757 | *Ae. tauschii* | + |  | 2 | 0.058 (2.23) | 0.011 (0.42) |
|  | AC237758 | *Ae. tauschii* | + |  | 2 | 0.055 (2.12) | 0.011 (0.42) |
|  | AC237760 | *Ae. tauschii* | + |  | 2 | 0.058 (2.23) | 0.011 (0.42) |
|  | AC237785 | *Ae. tauschii* | + |  | 2 | 0.037 (1.42) | 0.009 (0.35) |
|  | AY914086 | *T. aestivum* 5BL | + |  | 2 | 0.049 (1.88) | 0.010 (0.38) |
|  | DQ267103-1 | *T. aestivum* 5BL | + |  | 2 | 0.037 (1.42) | 0.009 (0.35) |
|  | DQ267103-2 | *T. turgidum* 4B | + |  | 2 | 0.029 (1.12) | 0.008 (0.31) |
|  | DQ157836 | *T. turgidum* 5BL | + |  | 2 | 0.020 (0.77) | 0.006 (0.23) |
|  | DQ157839 | *T. turgidum* 5BL | + |  | 2 | 0.020 (0.77) | 0.007 (0.27) |
|  | DQ157840 | *T. turgidum* 5BL | + |  | 2 | 0.020 (0.77) | 0.006 (0.23) |
|  | AM072971 | *T. aestivum* 5BL | + |  | NI |  |  |
|  | AM087557 | *T. aestivum* 5AL | + |  | NI |  |  |
|  | AM050679 | *T. aestivum* 5B | + |  | NI |  |  |
|  | AM050681 | *T. aestivum* 5BL | + |  | NI |  |  |
|  | GQ422824-1 | *T. aestivum* 3B | + |  | NI |  |  |
|  | GQ422824-2 | *T. aestivum* 3B | + |  | NI |  |  |
|  | GQ422824-3 | *T. aestivum* 3B | + |  | NI |  |  |
|  | GQ422824-4 | *T. aestivum* 3B | + |  | NI |  |  |
|  | GQ422824-5 | *T. aestivum* 3B | + |  | NI |  |  |
|  | HM775491 | *T. aestivum 7A* | + |  | NI |  |  |
|  | AC237686 | *Ae. tauschii* | + |  | NI |  |  |
|  | AC237688 | *Ae. tauschii* | + |  | NI |  |  |
|  | AC241775-1 | *Ae. tauschii* | + |  | NI |  |  |
|  | AC241775-2 | *Ae. tauschii* | + |  | NI |  |  |
|  | AC241768 | *Ae. tauschii* | + |  | NI |  |  |
|  | AC241765 | *Ae. tauschii* | + |  | NI |  |  |
|  | AC241774 | *Ae. tauschii* | + |  | NI |  |  |
|  | AC241761 | *Ae. tauschii* | + |  | NI |  |  |
|  | AC237773 | *Ae. tauschii* | + |  | NI |  |  |
|  | AC237774 | *Ae. tauschii* | + |  | NI |  |  |
|  | AC237741 | *Ae. tauschii* | + |  | NI |  |  |
|  | AC237744 | *Ae. tauschii* | + |  | NI |  |  |
|  | AC237753 | *Ae. tauschii* | + |  | NI |  |  |
|  | AC237717 | *Ae. tauschii* | + |  | NI |  |  |
|  | AC241753 | *Ae. tauschii* | + |  | NI |  |  |
|  | AC241759 | *Ae. tauschii* | + |  | NI |  |  |
| **Nonautonomous elements*** | | | | | | | |
| № | **Accession** | **Sequence is belong to species (genome or chromosome)** | **PTREP**  **231** | **PTREP**  **232** | **Number of LTR** | **LTR Divergence (Insertion time in MYA)** | **Standard error (time in MYA)** |
|  | TREP3190 | *T. aestivum* | + | + | 1 |  |  |
|  | TREP3191 | *T. aestivum* | + | + | 2 | 0.035 (1.35) | 0.009 (0.35) |
|  | TREP3192 | *T. aestivum* | + | + | 1 |  |  |
|  | TREP3193 | *T. aestivum* | + | + | 2 | 0.067 (2.58) | 0.012 (0.46) |
|  | TREP3194 | *T. aestivum* | + | + | 2 | 0.018 (0.69) | 0.006 (0.23) |
|  | TREP3195 | *T. aestivum* | + | + | 2 | 0.017 (0.65) | 0.006 (0.23) |
|  | TREP3196 | *T. aestivum* | + | + | 1 |  |  |
|  | TREP3197 | *T. aestivum* | + | + | 2 | 0.075 (2.88) | 0.014 (0.54) |
|  | TREP1804 (294D11-2) | *T. durum* | + | + | 2 | 0.013 (0.50) | 0.005 (0.19) |
|  | TREP1231(AY188332) (609E6-1) | *T. monococcum* | + | + | 1 |  |  |
|  | TREP2268 (DQ267105) (643D12-1) | *T. durum* (4A) | + | + | 2 | 0.052 (2.00) | 0.010 (0.38) |
|  | TREP2269 (DQ267105) (643D12-2) | *T. durum* (4A) | + | + | 1 |  |  |
|  | TREP235 (AF326781-1) | *T. monococcum* | + | + | 2 | 0.037 (1.42) | 0.009 (0.35) |
|  | TREP1306 (AY485644-2 ) | *T. monococcum* | + | + | 2 | 0.032 (1.23) | 0.008 (0.31) |
|  | TREP827 (AY146587-1) | *T. durum* | + | + | - |  |  |
|  | TREP1414 (AY494981-1) (634M12-1) | *T. turgidum* (A) | + | + | 2 | 0.032 (1.23) | 0.008 (0.31) |
|  | DQ537335-1 | *T. aestivum* (1A) | + | + | 2 | 0.032 (1.23) | 0.008 (0.31) |
|  | DQ537335-3 | *T. aestivum* (1A) | + | + | 1 |  |  |
|  | AM932686-1 (TA3B95G2-1) | *T. aestivum* (3B) | + | + | 2 | 0.028 (1.08) | 0.008 (0.31) |
|  | AM932686-2 (TA3B95G2-2) | *T. aestivum* (3B) | + | + | 2 | 0.019 (0.73) | 0.006 (0.23) |
|  | AM932686-3 (TA3B95G2-3) | *T. aestivum* (3B) | + | + | 2 | 0.044 (1.69) | 0.010 (0.38) |
|  | AM932686-5 (TA3B95G2-5) | *T. aestivum* (3B) | + | + | 2 | 0.055 (2.12) | 0.011 (0.42) |
|  | AY951944-1 (21C6-1) | *T. monococcum* | + | + | 2 | 0.046 (1.77) | 0.010 (0.38) |
|  | AY494981-3 (110M05-3) | *T. turgidum* (A) | + | + | 1 |  |  |
|  | AM932684-2 (TA3B95C9-2) | *T. aestivum* (3B) | + | + | 2 | 0.042 (1.62) | 0.010 (0.38) |
|  | AM932684-3 (TA3B95C9-3) | *T. aestivum* (3B) | + | + | 2 | 0.049 (1.88) | 0.010 (0.38) |
|  | AM932685-2 (TA3B95F5-2) | *T. aestivum* (3B) | + | + | 2 | 0.026 (1.00) | 0.008 (0.31) |
|  | AM932680-2 (TA3B54F7-2) | *T. aestivum* (3B) | + | + | 1 |  |  |
|  | AM932680-3 (TA3B54F7-3) | *T. aestivum* (3B) | + | + | 1 |  |  |
|  | EF426565-2 | *T. aestivum* | + | + | 1 |  |  |
|  | EF426565-3 | *T. aestivum* | + | + | 2 | 0.035 (1.35) | 0.009 (0.35) |
|  | AY663391-1 | *T. turgidum* | + | + | 2 | 0.022 (0.85) | 0.007 (0.27) |
|  | AH012974-1 (AF532104S1) | *Ae. tauschii* | + | + | 1 |  |  |
|  | AY188333-2 (719C13-2) | *T. monococcum* (5AL) | + | + | 1+fr |  |  |
|  | EU660902-1 | *T. aestivum* (2DS) | + | + | 1+fr |  |  |
|  | EU660896-2 | *T. urartu* (2AS) | + | + | 2 | 0.028 (1.08) | 0.008 (0.31) |
|  | EU660898-1 | *T. turgidum* (2AS) | + | + | 2 | 0.034 (1.31) | 0.009 (0.35) |
|  | EU660894-1 | *T. turgidum* (3BL) | + | + | 1+fr |  |  |
|  | EF081026-1 | *T. turgidum* (4A) | + | + | 2 | 0.048 (1.85) | 0.011 (0.42) |
|  | EU660896-5 | *T. urartu* (2AS) | + | + | 2 | 0.036 (1.38) | 0.008 (0.31) |
|  | EU660896-3 | *T. urartu* (2AS) | + | + | 2 | 0.041 (1.58) | 0.009 (0.35) |
|  | CT009625-1 | *Ae. tauschii* (5D) | + | – | 1 |  |  |
|  | AY368673-2 (107M9-3) | *T. turgidum* (1B) | + | – | 1 |  |  |
|  | EF426565-1 | *T. aestivum* | + | – | 1 |  |  |
|  | EU660897-1 | *Ae. tauschii* (2S) | + | – | 1 |  |  |
|  | TREP828 (AY146587-2) | *T. durum* | + | – | 1 |  |  |
|  | TREP252 (AF459639) (116F2-1) | *T. monococcum* (5A) | + | – | 1 |  |  |
|  | AB298185-1 | *T. aestivum* (5BS) | + | – | 1 |  |  |
|  | TREP2267 (210J24-1) | *T. urartu* | - | + | 1 |  |  |
|  | TREP2209 (464G14-1) | *T. aestivum* | - | + | 1 |  |  |
|  | AM932684-1 (TA3B95C9-1) | *T. aestivum* (3B) | - | + | 1 |  |  |
|  | AM932682-1 (TA3B63B7-1) | *T. aestivum* (3B) | - | + | 1 |  |  |
|  | EF567062-1 | *T. aestivum* | - | + | 1 |  |  |
|  | EF426565-4 | *T. aestivum* | - | + | 1 |  |  |
|  | AY485644-1 | *T. monococcum* | - | + | 1 |  |  |
|  | EU660896-1 | *T. urartu* (2AS) | - | + | – |  |  |
|  | EU660896-6 | *T. urartu* (2AS) | - | + | 1 |  |  |
|  | EU660900-1 | *T. aestivum* (2AS) | - | + | 1 |  |  |

Notes: The single element is denoted as TREP accession, or accession of GenBank genomic sequence from where the element was taken of. In the last case the numbering after accession indicate the ordinal number of Fatima element in genomic sequence. The columns PTREP231–PTREP234 reflect the presence (+) or absence (–) of corresponding coding capacity in elements. PTREP231 and PTREP233 are the consensus polyprotein aminoacid sequences for nonautonomous and autonomous *Fatima* elements, respectively, from the TREP hypothetical protein database; PTREP232 and PTREP234 are the consensus aminoacid sequences for “non-autonomous” and “autonomous” additional Fatima ORF. The presence of coding sequences homologous to hypothetical PTREP231 and/or PTREP232 imply the element accessory to “non-autonomous” subfamily, and PTREP233 and/or PTREP234 to “autonomous”.

NI – LTR not identified.

*The data for 58 from 165 “non-autonomous” identified elements are presented.
